# Data-driven causal model discovery and personalized prediction in Alzheimer's disease

**DOI:** 10.1038/s41746-022-00632-7

**Published:** 2022-09-08

**Authors:** Haoyang Zheng, Jeffrey R. Petrella, P. Murali Doraiswamy, Guang Lin, Wenrui Hao, for the Alzheimer’s Disease Neuroimaging Initiative

**Affiliations:** 1 School of Mechanical Engineering, Purdue University, West Lafayette, 47907 IN USA; 2 Department of Radiology, Duke University Health System, Durham, 27710 NC USA; 3 Departments of Psychiatry and Medicine, Duke University School of Medicine and Duke Institute for Brain Sciences, Durham, 27710 NC USA; 4 Department of Mathematics, Purdue University, West Lafayette, 47907 IN USA; 5 Department of Mathematics, Penn State University, University Park, 16802 PA USA

**Keywords:** Computational models, Data processing

## Abstract

With the explosive growth of biomarker data in Alzheimer’s disease (AD) clinical trials, numerous mathematical models have been developed to characterize disease-relevant biomarker trajectories over time. While some of these models are purely empiric, others are causal, built upon various hypotheses of AD pathophysiology, a complex and incompletely understood area of research. One of the most challenging problems in computational causal modeling is using a purely data-driven approach to derive the model’s parameters and the mathematical model itself, without any prior hypothesis bias. In this paper, we develop an innovative data-driven modeling approach to build and parameterize a causal model to characterize the trajectories of AD biomarkers. This approach integrates causal model learning, population parameterization, parameter sensitivity analysis, and personalized prediction. By applying this integrated approach to a large multicenter database of AD biomarkers, the Alzheimer’s Disease Neuroimaging Initiative, several causal models for different AD stages are revealed. In addition, personalized models for each subject are calibrated and provide accurate predictions of future cognitive status.

## Introduction

Among the top 10 causes of death in the United States, Alzheimer’s disease (AD) is the only condition without a viable treatment to cure or prevent it, or even significantly slow its progression^1^. Failure to develop a successful disease-modifying therapy for AD, despite large investments of public and private resources, is rooted in its complexity^2–4^. For instance, signaling pathway analyses of AD pathophysiology has implicated over 30 metabolic pathways and over 1000 chemical species^4^. Our incomplete understanding of how these mechanisms vary and interact at an individual level to create a clinically and biologically heterogeneous phenotype has resulted in an attempt to treat patients with varying underlying pathophysiology in a similar fashion^5,6^. Thus, failure to characterize and subtype AD at an individual level has represented a major roadblock in the development of effective therapeutic strategies to slow or halt AD progression. Recent biological classification of AD, based on imaging and cerebral spinal fluid (CSF) biomarkers, represents a major step toward the future development of personalized prognoses and therapeutic strategies^7,8^. The increasing availability of such data in large cohorts of subjects has made possible the development and testing of rigorous quantitative models of AD pathophysiology. For example, the Alzheimer’s Disease Neuroimaging Initiative (ADNI), a multicenter, prospective, naturalistic study, began in 2003, comprises four sequential studies—ADNI-1, ADNI-GO, ADNI-2, and ADNI-3—which followed subjects up to 15 years, using genetic, blood- and CSF-based, imaging, and cognitive biomarkers. The abundance of data from this and similar multinational biomarker studies in AD will require a rigorous quantitative data-driven modeling approach to analyze, integrate and interpret data at the level of the individual, where it can have maximum clinical impact.

Several mathematical models of AD progression have been developed recently. For example, one mathematical model includes a cellular biologic system of neurons, glia, macrophages, amyloid*β* aggregation, and tau to simulate and validate at a cellular level the mechanisms underlying the failure of several drugs in recent clinical trials, and suggest alternative approaches^9^. Moreover, a mathematical modeling approach has also been used to describe the key AD clinical biomarkers including pathologic hallmark biomarkers (beta-amyloid and tau), neuronal loss biomarkers, and cognitive impairment^10^. This model was parameterized and tested to successfully simulate the natural history scenarios of three sub-types of AD presented in^11^: (1) early-onset autosomal dominant AD, (2) late-onset amyloid-first AD, and (3) late-onset tau-first AD.

Although these mathematical models bring new insights in understanding AD progression and enable simulation of therapeutics, the current models are built upon a priori hypotheses of the AD pathophysiological network which still is an open area of research^12^. In fact, there are dozens of pathophysiological pathways implicated in AD by systems biologists, and our understanding of these networks and their interactions remains incomplete^13^. Moreover, there has been limited work on mechanistic modeling of clinically measurable AD biomarkers. Most research to date on the keyAD biomarkers has been observational or correlational. Such modeling approaches do not benefit from the tools of a more integrative systems approach that address disease mechanism^14,15^.

Computational data-driven modeling approaches have already achieved success in analyzing multi-dimensional clinical data in diseases such as cancer^16,17^ and cardiovascular disease^18,19^. Such data-driven approaches employ mathematical models for patient populations using clinical, omics, and biomarker data, as well as powerful and new means to personalize such models based on individual data, yielding personal risk profiles. These data-driven modeling approaches can simulate complex systems, helping to elucidate complex physiological interactions and optimize personalized prevention and treatment strategies. Examples of such work include statistical approaches, such as Bayesian generalized linear models^20^, Bayesian hierarchical models^21,22^, and those based on Markov chain Monte Carlo simulations^23–26^ to analyze genome sequencing and biomarker dynamics. Recently, machine learning techniques, such as deep recurrent neural networks, have been used to predict AD progression^27^.

In this paper, we propose to develop a computational data-driven modeling framework to predict AD biomarker progression. We propose a methodology to construct data-driven causal models at a group and individual patient level. This method does not depend on any specific hypothesis of AD progression and extracts the causal model completely from the empirical data. More specifically, we derive the causal model based on clinical biomarkers in the ADNI dataset. In this data-driven modeling approach, the causal model is learned from four biomarkers (amyloid-beta pathology, total-tau pathology, hippocampal volume, and cognitive decline) to describe AD progression. Moreover, we incorporate a disease progression score (DPS) in the causal model^28^ to unify AD progression for different subjects since the onset age and rates of progression may markedly vary within and across the different subject classes in ADNI.

## Results

We elaborate on the effectiveness of the proposed data-driven causal model here. First, we construct a population-based causal model that describes the biomarker dynamics for all eligible subjects in ADNI-1, including normal controls. By fitting the population parameters via the ADNI dataset, the population model describes the transition of AD biomarkers between three different disease stages, cognitively normal (CN), late mild cognitive impairment (LMCI), and Alzheimer’s disease (AD). Second, we derive a population model for LMCI and AD subjects only. Third, we analyze the Sobol sensitivity^29,30^ of the parameter space of the population model, which identifies the attribution of each model parameter. Based on the sensitivity analysis results, we finally construct a personalized model for each subject and provide personalized biomarker predictions for subjects who have more than four longitudinal biomarker data points.

### A population model

**Fig. 1:**
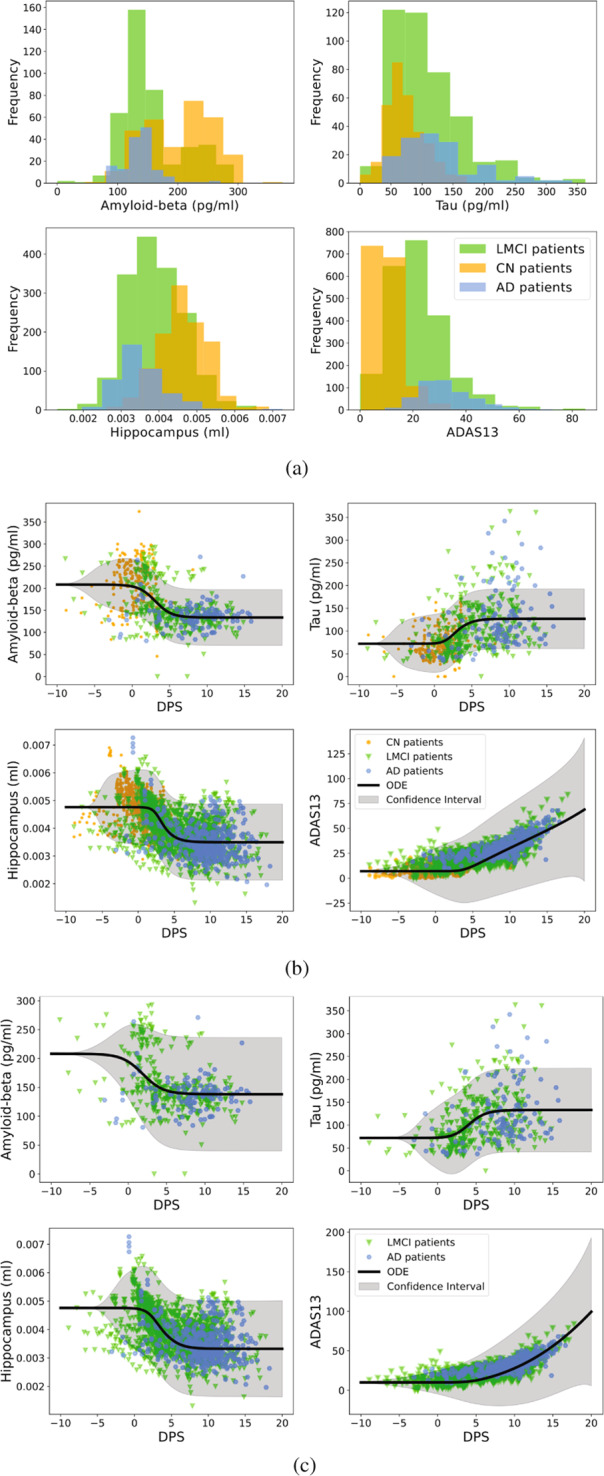
The ADNI dataset histogram and the calibrated causal model plots. **a** Histogram of four biomarkers in the ADNI dataset. Top left is amyloid-beta; top right is the tau; bottom left is the normalized hippocampal volume; bottom right is the cognitive subscale. X-axes are the corresponding magnitudes of each biomarker, and y-axes are their frequencies. The subjects are classified into “CN”, “LMCI”, and “AD”. **b** The calibrated causal model on three groups of patient data. X-axes are fitted DPS of biomarkers, and y-axes are the corresponding magnitudes of each biomarker. The orange circles, green triangles, and blue hexagons are data from “CN”, “LMCI”, and “AD” subjects correspondingly. The black solid lines are the solutions of the causal model. **c** The calibrated causal model on the dataset of LMCI and AD groups.

We construct a causal model by fitting biomarkers of all subjects across the ADNI dataset. Since the causal model is a dynamic system expressed as ordinary differential equations (ODEs), we require at least two longitudinal data points for each subject. More specifically, we remove patients who do not provide at least two measurements for any one of the four biomarkers. The histograms in Fig. 1(a) summarize the available biomarker data in the ADNI dataset.

#### Algorithm 1

Population model calibration algorithm to compute the population parameters ***w***^(1)^ and DPS parameters (***α***, ***β***). See details in Methods section.

**Input** $${{{\boldsymbol{y}}}}={\{{y}_{ijk}\}}_{ijk}$$, $${{{\boldsymbol{t}}}}={\{{t}_{ij}\}}_{ij}$$.

**Initialize** ***α***^0^, ***β***^0^, and ***w***^0^.

1: **for** *l*=1 to *L* **do**

2: **for** *k* ∈ {*A*, *T*, *N*, *C*} **do** ⊳ Population parameter calibration

3: $${{{{\boldsymbol{w}}}}}_{k}^{l}={{{\mbox{argmin}}}}_{{{{\boldsymbol{{w}}}_{k}}}}{\sum }_{(i,j)\in {{{{\mathcal{I}}}}}_{k}}{\left({y}_{ijk}-{f}_{k}\left({\alpha }_{i}^{l}{t}_{ij}+{\beta }_{i}^{l};{{{{\boldsymbol{w}}}}}_{k}^{l-1}\right)\right)}^{2}.$$

4: $${\sigma }_{k}^{l}=\frac{1}{| {{{{\mathcal{I}}}}}_{k}-2I-4| }{\sum }_{(i,j)\in {{{{\mathcal{I}}}}}_{k}}{\left({y}_{ijk}-{f}_{k}\left({\alpha }_{i}^{l}{t}_{ij}+{\beta }_{i}^{l};{{{{\boldsymbol{w}}}}}_{k}^{l-1}\right)\right)}^{2}.$$

5: **end** **for**

6:

7: **for** *i*=1 to *I* **do** ⊳ Update DPS parameters

8: $$({\alpha }_{i}^{l},\,{\beta }_{i}^{l})={{{\mbox{argmin}}}}_{{\alpha }_{i},{\beta }_{i}}{\sum }_{(j,k)\in {{{{\mathcal{I}}}}}_{i}}\frac{1}{{\sigma }_{k}^{l}}{\left({y}_{ijk}-{f}_{k}\left({\alpha }_{i}^{l}{t}_{ij}+{\beta }_{i}^{l};{{{{\boldsymbol{w}}}}}_{k}^{l}\right)\right)}^{2}.$$

9: **end for**

10: **end for**

**Output** ***w***^*L*^ as the population parameter ***w***^(1)^, ***α***^*L*^, ***β***^*L*^.

By using Algorithm 1, the initial value of ***α***_*i*_ is randomly chosen in (0, 4) and the initial value of ***β***_*i*_ satisfies − 10 ≤ *s*_*i*_(*t*) ≤ 20 on all the measurement. Then we obtain the population model in terms of the fitted DPS shown in Fig. 1(b). The population model (black solid) is learned on three different disease stages, namely, CN (orange circle), LMCI (green circle), and AD (blue hexagons). The gray area is the confidence interval of the population model. More specifically, we sample the population parameters, ***w***^(1)^, from the posterior distribution (given by the simulation study) and run the model with the same initial condition 1000 times. Then the 95% confidence interval at every time point is plotted. The simulation study and diagnostic plots corresponding to the population model are shown in the Supplementary Materials. From this figure, we can separate biomarkers into three stages according to the population model. In particular, CN and AD patients correspond to *s* < 0 and *s* > 0, respectively while LMCI patients locate around *s* = 0. Moreover, the first three biomarkers (*A*_*β*_, *τ*, and *N*) start at steady-states when *s* < 0 (CN), change gradually when *s* = 0 (LMCI), and finally approach another steady-state (AD). Different from other biomarkers, ADAS continues to grow which means that cognitive symptoms get worse as AD progresses.

**Fig. 2:**
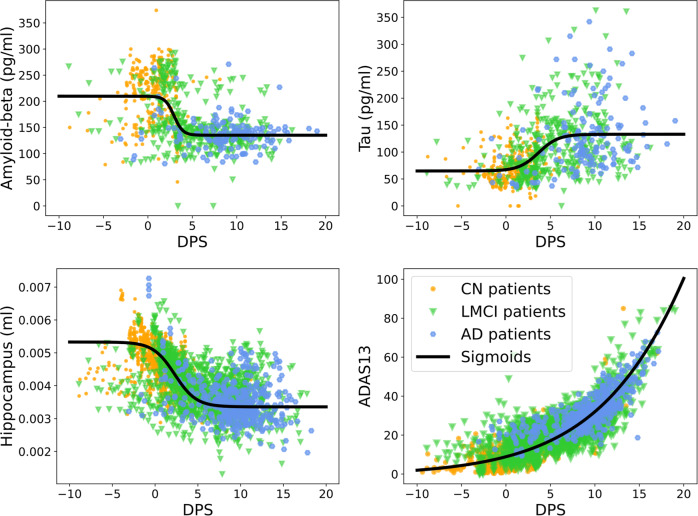
Subject biomarkers in ADNI data and the fitted sigmoid function. Top left is amyloid-beta; top right is the tau; bottom left is the normalized hippocampal volume; bottom right is the cognitive subscale. X-axes are fitted DPS of biomarkers, and y-axes are the corresponding magnitudes of each biomarker. The subjects are classified into “CN”, “LMCI”, and “AD” according to ADNI diagnostic groups, where orange is “CN”, green is “LMCI”, and blue is “AD”. The black solid lines are fitted with sigmoid functions^28^.

We also compare the population model with the sigmoid function fitting (black solid in Fig. 2). First, the population model provides relatively smooth transitions from one stage to another while the sigmoid function fitting gives more abrupt changes for *A*_*β*_ at *s* ≈ 4. Second, the population model follows the biomarker cascade theory which is that *τ* rises after *A*_*β*_ starts decreasing, *N* increases after *τ*, and *C* rises after *N*. However, the sigmoid function fitting makes *A*_*β*_ and *τ* change after *s* = 0, while *N* and *C* change at *s* ≈ −3 and *s* ≈ −10.

Since the CN group might not follow the same disease trajectory, we next derive a population model based on the LMCI and AD groups only. In order to better identify the biomarker dynamics among LMCI and AD group patients, we fix the parameters of DPS, (***α***, ***β***), that we obtained before and only update the causal model parameters, ***w***, by using Algorithm 1. Figure 1(c) shows the population model based on LMCI and AD groups.

The transitions for different biomarkers shown in Fig. 1(c) are similar to Fig. 1(b). But Fig. 1(c) advances the onset of changes since the LMCI and AD groups are prone to suffer from cognitive decline earlier. We summarize the parameters of the above-mentioned causal models in Table 1, which corresponds to the results given in Fig. 1(b) & (c).

**Table 1:** Population parameters ***w***^(1)^ of the calibrated causal models based on the ADNI dataset.

Biomarkers	Parameters	Included subjects	
		CN, LMCI, AD	LMCI, AD
***A***_*β*_	*w*_*A*0_	0	0
	*w*_*A*1_	0.917	0.745
	*w*_*A*2_	–0.873	–0.749
***τ***	*w*_*T*0_	0	0
	*w*_*T*1_	0.788	0.689
	*w*_*T*2_	–0.246	–0.679
	*w*_*T*3_	0.002	0.000
	*w*_*T*4_	3.066	0.185
	*w*_*T*5_	–3.650	–0.101
***N***	*w*_*N*0_	0	0
	*w*_*N*1_	1.627	0.899
	*w*_*N*2_	–1.253	–0.927
	*w*_*N*3_	0.018	0.554
	*w*_*N*4_	2.342	1.792
	*w*_*N*5_	–4.015	–2.127
***C***	*w*_*C*0_	0	0
	*w*_*C*1_	0.159	0.134
	*w*_*C*2_	0.202	–0.067
	*w*_*C*3_	0.010	0.004
	*w*_*C*4_	0.019	0.007
	*w*_*C*5_	–0.176	–0.008
Initial conditions	*y*_0_	6.35e–6	1.41e–4

Superscripts of parameters are omitted from the table. See details in Methods section.

### Sensitivity analysis

**Fig. 3:**
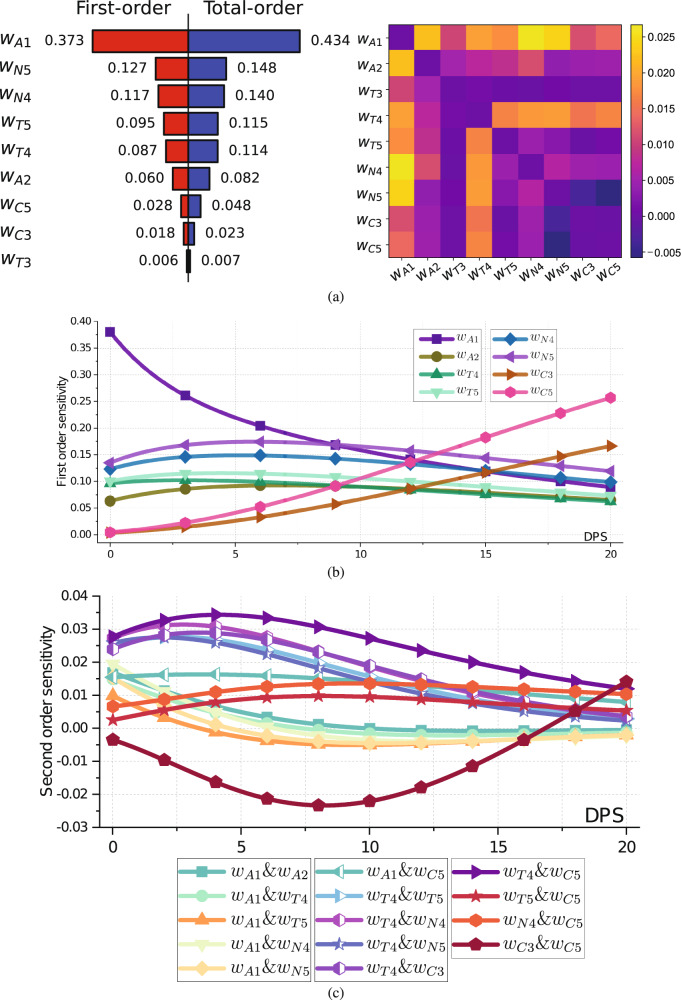
The Sobol sensitivity analysis plots with respect to cognitive decline. **a** First-order, second-order, and total-order Sobol sensitivities of *C*(0). Left: the red rectangles are assigned first-order sensitivities of model parameters, and the blue rectangles are their total-order sensitivities. The length of the rectangles represents the attribution of sensitivities to outputs; Right: each square represents the second-order sensitivity correlations of two model parameters. The lighter the color, the stronger the positive correlation while the darker the color, the stronger the negative correlation. **b** The dynamics of first-order Sobol sensitivities with respect to DPS. Each curve corresponds to the first-order sensitivity values with an output *C*(*s*). Only first-order sensitivity values greater than 0.01 are plotted. **c** The dynamics of second-order Sobol sensitivities for two parameters with respect to DPS. Only the maximum absolute second-order sensitivity values greater than 0.01 are plotted.

The quasi-Monte Carlo method is applied to compute sensitivity indices. For more details about Sobol sensitivity analysis, please refer to^29,30^. By taking *C*(0) as the output, Fig. 3(a) list the top nine most sensitive parameters for the first-order effects and total order sensitivity index. We see that the weight with greater first-order impact (*S*_*m*_ > 0.4) is associated with *A*_*β*_(⋅).

The right figure in 3(a) shows the second-order interaction between two parameters. We see that the parameters associated with ${A}_{\beta }^{2}$ are always positively related to other terms. While *A*_*β*_ with parameter *w*_*A*1_ is almost positively related with other terms, the *A*_*β*_ term with parameter *w*_*T*3_ are negatively related with other parameters except ${A}_{\beta }^{2}$. But compared to their first-order sensitivity contribution, the second-order ones contribute slightly.

Figure 3(b, c) shows the dynamics of sensitivities with respect to DPS. From the figures, we can see the first-order sensitivity value of *w*_*A*1_ drops down over DPS which implies that the effect of Abeta on cognitive decline switches from linear to nonlinear in later-stage disease. At the same time, the first-order sensitivity values of some other parameters increase gradually, with a notable increase of *w*_*C*3_ and *w*_*C*5_. The second-order sensitivities between different parameters eventually converge to zero thus the interactions among different parameters become less as the biomarkers reach equilibrium. Based on the results shown in Fig. 3(a), we select *w*_*A*1_, *w*_*A*2_, *w*_*T*4_, *w*_*T*5_, *w*_*N*4_, *w*_*N*5_, *w*_*C*3_, and *w*_*C*5_ as the most sensitive parameters for personalization by setting the threshold, Tol, as 0.01 in Algorithm 2.

### Personalized model and biomarker prediction

#### Algorithm 2

Personalized model calibration algorithm. The personalized parameters are initialized by the population model. The personalized models are applied for subjects who meet the requirement denoted as *i* ∈ Ω.

**Input** longitudinal biomarker data {*y*_*ijk*_} at {*t*_*ij*_} with *i* ∈ Ω;

**Input** the DPS parameter values (*α*_*i*_, *β*_*i*_) for each subject *i* ∈ Ω;

**Input** the population parameter values ***w***^(1)^ (***w*** for simplicity);

**Input** sensitivity threshold, TOL.

1: **for** *m*=1 to 21 **do** ⊳ First order sensitivity.

2: $${{{{\rm{S}}}}}_{m}(z)=\frac{{{{{\rm{Var}}}}}_{{w}_{m}}\left[{{{{\rm{E}}}}}_{{{{\rm{{w}}}_{ \sim m}}}}(z| {w}_{m})\right]}{{{{\rm{Var}}}}(z)}.$$

3: **if** *S*_*m*_(*z*)≥ TOL **then**

4: set *w*_*m*_ as a personalized parameter and denote as ${w}_{m}^{(2)}$ else

5: keep *w*_*m*_ as a population parameter.

6: **end** **if**

7: **end** **for**

8:

9: **for** *i*=1 to ∣Ω∣ **do** ⊳ Personalized model calibration.

10: **for** *k* ∈ {*A*, *T*, *N*, *C*} **do**

11: Denote the personalized parameters in *k*-th equation as ${{{{\boldsymbol{{w}}}_{k}}}}^{(2)}$.

12: ⊳ Select parameters to calibrate.

13: $${{{{\boldsymbol{{w}}}_{k}}}}^{(2)}={\arg \min }_{{{{{\boldsymbol{{w}}}_{k}}}}^{(2)}}\mathop{\sum }\limits_{j=1}^{M-1}{\left({\hat{y}}_{ijk}-{f}_{k}\left({\alpha }_{i}{t}_{ij}+{\beta }_{i};{{{{\boldsymbol{{w}}}_{k}}}}^{(2)}\right)\right)}^{2}.$$

14: $$P{A}_{ik}=\frac{{\hat{y}}_{iMk}-{f}_{k}\left({\alpha }_{i}{t}_{(iM)}+{\beta }_{i};{{{{\boldsymbol{{w}}}_{k}}}}^{(2)}\right)}{{\hat{y}}_{iMk}}\times 100 \% .$$

15: ⊳ Compute prediction accuracy.

16: **end for**

17: **end for**

**Output** *PA*_*ik*_ for *i* ∈ Ω and *k* ∈ {*A*, *T*, *N*, *C*}.

Next, we build personalized models and provide biomarker prediction for subjects whose data satisfies the following two criteria: (1) There are at least four measurements for each biomarker; (2) Each biomarker measurement changes monotonically with respect to DPS. Based on the first-order sensitivity analysis results shown in Fig. 3(a), we chose the eight most sensitive parameters as personalized parameters by choosing TOL = 0.01 in Algorithm 2. For each subject, we denote the biomarker data as $\hat{{{{\boldsymbol{y}}}}}({s}_{i})={[{\hat{{{{\boldsymbol{A}}}}}}_{\beta }({s}_{i})\hat{{{{\boldsymbol{\tau }}}}}({s}_{i})\hat{{{{\boldsymbol{N}}}}}({s}_{i})\hat{{{{\boldsymbol{C}}}}}({s}_{i})]}^{T}$ (*i* = 1, ⋯ , *M*), fit the sensitive personalized parameters of the population model ***w***^(1)^ by using the first *M* − 1 data points, and test the prediction accuracy on the last data point by $\frac{\hat{{{{\boldsymbol{y}}}}}({s}_{M})-{{{\boldsymbol{y}}}}({s}_{M})}{\hat{{{{\boldsymbol{y}}}}}({s}_{M})}\times 100 \%$. A detailed procedure is outlined in Algorithm 2.

**Fig. 4:**
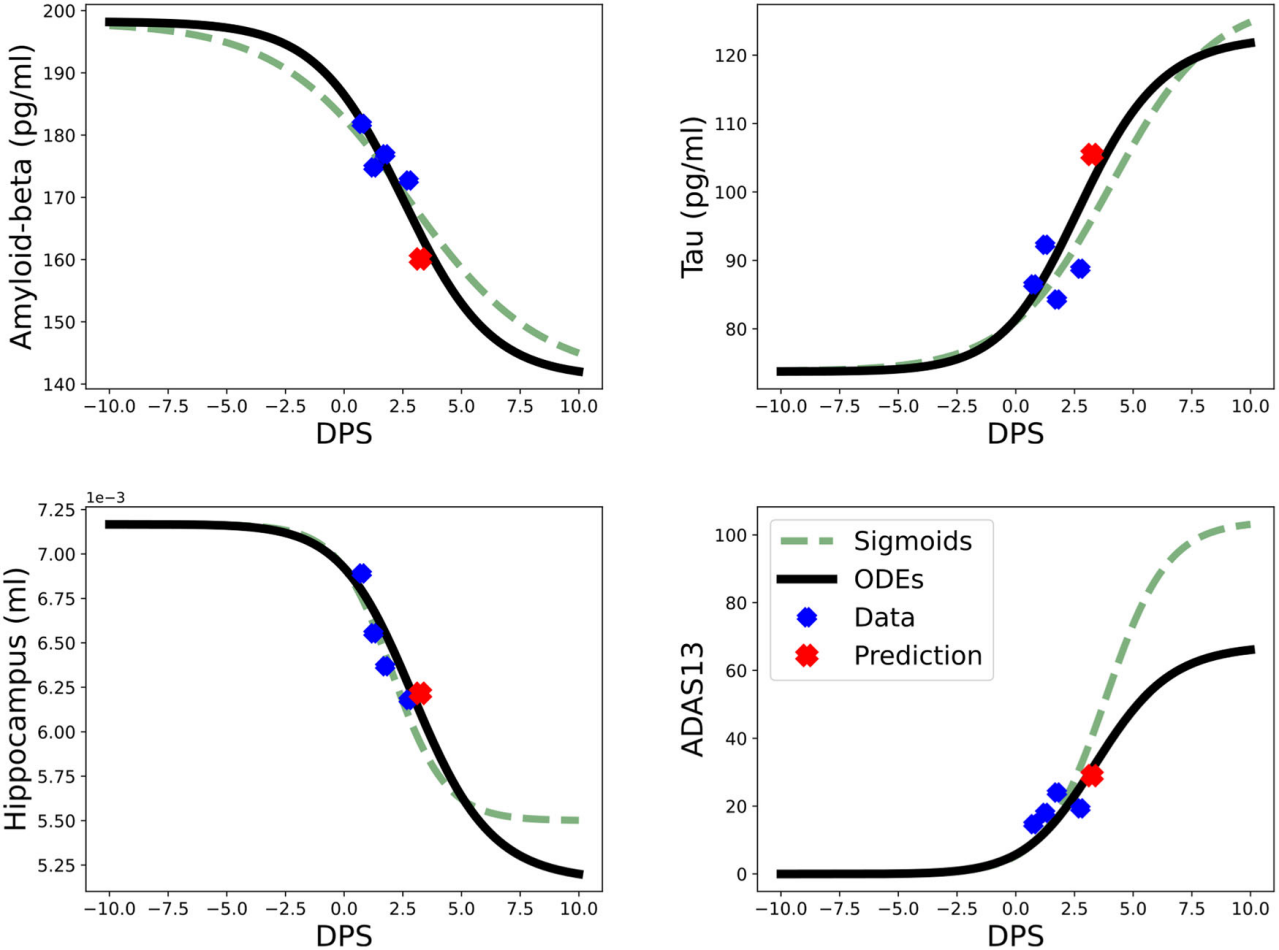
The personalized model for one LMCI subject with pseudo ID = 18. The green dashed lines are fitted by sigmoid functions, and black solid lines are the solutions of the personalized model. The blue markers are training data points while the red markers are for testing.

Figure 4 shows the biomarker trajectories of the personalized model by training (blue) and testing (red) data for one subject (pseudo ID = 18). We also compare the personalized model with the sigmoid function fitting, the personalized model provides a better prediction accuracy. In fact, the prediction accuracies given by the personalized model are 97.3% (*A*_*β*_), 95.9% (*τ*), 98.4% (*N*), and 95.1% (*C*), respectively while the ones given by the sigmoid function fitting are 95.5% (*A*_*β*_), 90.8% (*τ*), 95.7% (*N*), and 63.4% (*C*), respectively. Since the sigmoid function fitting predicts by using the longitudinal information of the current biomarker only, it provides a less accurate cognitive score.

Furthermore, we build personalized models for the CN and LMCI groups (there are not enough data points in the AD group) with different numbers of longitudinal data points and summarize the predictive results in Tables 2–3. The tables indicate that our personalized models can provide high predictive accuracy compared to the sigmoid function fitting. Moreover, the accuracy of predicting biomarker dynamics increases as the number of biomarkers data points increases.

**Table 2:** The prediction accuracy summary for CN subjects using different numbers of longitudinal biomarker datapoints (*n*).

PseudoIDs (n)	DPS Diff	Model	Accuracy			
			CSF Abeta42	CSF tTau	HIPPv	ADAS13
1 (4)	0.13	ODE	98.3%	93.6%	99.4%	92.6%
		Sigmoid	74.0%	79.8%	70.5%	84.8%
2 (4)	3.00	ODE	99.8%	93.2%	98.7%	93.0%
		Sigmoid	93.9%	61.5%	90.7%	80.4%
3 (5)	0.52	ODE	86.6%	98.8%	95.9%	85.3%
		Sigmoid	90.3%	82.6%	71.1%	56.9%
4 (5)	0.59	ODE	98.8%	96.1%	88.3%	96.6%
		Sigmoid	76.8%	76.9%	86.1%	66.7%
5 (5)	0.39	ODE	97.8%	90.0%	99.7%	94.8%
		Sigmoid	84.3%	79.5%	79.9%	81.9%
6 (4)	0.46	ODE	96.3%	93.6%	90.9%	92.7%
		Sigmoid	75.4%	91.1%	91.2%	84.0%
7 (4)	0.55	ODE	99.8%	88.2%	98.7%	90.3%
		Sigmoid	96.5%	86.0%	92.0%	72.3%
8 (4)	0.63	ODE	95.9%	98.9%	92.0%	92.6%
		Sigmoid	85.8%	86.8%	91.7%	96.6%
9 (4)	0.71	ODE	99.6%	96.1%	97.1%	87.5%
		Sigmoid	89.4%	80.3%	79.2%	69.5%
10 (5)	1.04	ODE	83.4%	81.2%	98.7%	85.5%
		Sigmoid	88.3%	78.4%	74.4%	80.1%
11 (6)	1.04	ODE	98.2%	99.8%	86.5%	85.1%
		Sigmoid	75.7%	76.3%	67.6%	72.6 %
12 (4)	0.40	ODE	94.6%	91.3%	96.5%	91.7%
		Sigmoid	89.7%	81.5%	88.9%	75.1%
13 (6)	0.88	ODE	97.0%	92.8%	96.1%	98.8%
		Sigmoid	97.4%	85.4%	85.3%	84.3%
14 (4)	0.75	ODE	98.4%	99.1%	99.1%	87.1%
		Sigmoid	90.9%	79.7%	88.6%	79.8%
15 (4)	0.55	ODE	99.6%	96.8%	90.9%	81.5%
		Sigmoid	93.8%	95.1%	81.5%	59.2%
Average	0.78 ± 0.64	ODE	96.3% ± 4.9%	94.0% ± 5.0%	95.2% ± 4.4%	90.3% ± 4.8%
		Sigmoid	86.8% ± 7.6%	81.4% ± 7.4%	82.6% ± 8.2%	76.3% ± 10.1%

The last four columns list the prediction accuracy of both the personalized model and the sigmoid function fitting for each biomarker. The last four rows summarize the mean and the standard deviation of prediction accuracy.

**Table 3:** The prediction accuracy summary for LMCI subjects using different longitudinal data points (shown in the first column).

PseudoIDs (n)	DPS Diff	Model	Accuracy			
			CSF Abeta42	CSF tTau	HIPPv	ADAS13
16 (5)	0.70	ODE	99.0%	87.8%	99.1%	88.2%
		Sigmoid	91.2%	87.4%	74.5%	70.1%
17 (6)	0.51	ODE	97.8%	86.2%	92.2%	90.7%
		Sigmoid	94.1%	83.3%	81.6%	88.7%
18 (5)	0.50	ODE	97.3%	95.9%	98.4%	95.1%
		Sigmoid	95.5%	90.8%	95.7%	63.4%
19 (4)	0.55	ODE	88.9%	96.3%	82.8%	96.6%
		Sigmoid	79.1%	87.7%	94.3%	85.1%
20 (5)	0.30	ODE	96.8%	96.7%	97.0%	84.9%
		Sigmoid	80.3%	89.1%	80.1%	82.1%
21 (6)	0.75	ODE	95.3%	85.8%	98.3%	84.4%
		Sigmoid	86.3%	72.9%	90.5%	79.8%
22 (4)	0.82	ODE	97.0%	95.7%	89.8%	84.6%
		Sigmoid	72.5%	68.5%	96.6%	81.6%
23 (4)	0.43	ODE	99.2%	91.0%	94.2%	86.0%
		Sigmoid	88.8%	61.3%	80.8%	62.1%
24 (6)	0.49	ODE	98.2%	98.9%	99.9%	96.9%
		Sigmoid	83.5%	89.9%	88.8%	81.1%
25 (6)	0.70	ODE	97.0%	94.4%	93.3%	89.3%
		Sigmoid	93.2%	80.2%	74.1%	70.7%
26 (4)	0.98	ODE	97.7%	93.5%	96.7%	93.8%
		Sigmoid	85.2%	89.1%	91.8%	81.3%
27 (4)	0.38	ODE	96.4%	97.4%	94.4%	96.3%
		Sigmoid	97.9%	79.5%	92.1%	72.6%
28 (5)	0.52	ODE	97.0%	90.6%	87.7%	96.6%
		Sigmoid	85.1%	78.4%	84.6%	83.1%
29 (4)	0.50	ODE	94.2%	91.4%	91.3%	95.0%
		Sigmoid	76.3%	82.7%	75.1%	72.1%
30 (4)	0.57	ODE	94.7%	90.0%	93.6%	83..1%
		Sigmoid	73.1%	80.6%	86.3%	85.8%
Average	0.58 ± 0.17	ODE	97.1% ± 1.5%	92.3% ± 4.1%	94.8% ± 3.6%	89.8% ± 5.4%
		Sigmoid	85.5% ± 7.8%	81.4% ± 8.2%	85.8% ± 7.5%	77.3% ± 7.9%

The last four columns list the prediction accuracy of both the personalized model and the sigmoid function fitting for each biomarker. The last four rows summarize the mean and the standard deviation of prediction accuracy.

## Discussion

Different from the existing pathophysiological AD network which is based on a priori assumptions about biomarker trajectories, this work develops a data-driven causal modeling approach informed by AD clinical biomarker data and demonstrates both population and personalized models. The proposed population model traces the general biomarker dynamics for all patient data without any specific assumptions regarding the form of the model and enables personalized AD risk prediction via incorporating historical clinical data such as CSF protein and imaging biomarkers as well as cognitive scores. By introducing a DPS for each subject, we calibrate and scale AD biomarker progression across the ADNI population and derive population parameters. We also compare the proposed data-driven modeling approach to an empirical fitting approach with a sigmoid function fitting and conclude that the proposed causal model is able to better capture disease progression with a smoother transition over time. Moreover, this causal model allows us to explore the underlying cascade relationship among biomarkers, while the empirical sigmoid function approach considers each biomarker as an independent term. The population model not only provides a means to classify different stages of AD progression for each biomarker, but also lays the foundation for personalized modeling.

Before constructing the personalized model, we performed a sensitivity analysis for the population parameters. From a clinical standpoint, the sensitivity analysis provides insights on AD progression in terms of which parameters play the greatest role in disease progression, and when during the disease course they are most relevant. From a computational standpoint, the sensitivity analysis aids the subsequent personalized parameter selection . Based on the sensitivity analysis, we see that change in cognition is driven primarily by first-order effects and is time-dependent. Initially, the greatest effects are by amyloid, represented by *w*_*A*1_, and to a lesser extent tau and neuronal vulnerability to tau, represented by *w*_*N*4_ and *w*_*N*5_, respectively. The amyloid parameter *w*_*A*1_ is most sensitive when the disease starts (*DPS* = 0) and the sensitivity diminishes as DPS increases. On the other hand, the sensitivity of parameters related to *N* and *C*, namely *w*_*C*3_ and *w*_*C*5_, increase significantly as the disease progresses. Thus, the sensitivity analysis suggests that at the early stage of AD cognitive decline is driven by *A*_*β*_ levels and sensitivity decreases linearly as the disease progresses. Whereas at the later stages, cognitive decline is driven mainly by downstream effects including the level of neuronal degeneration, represented by *w*_*C*3_, and the interaction of cognition and neuronal degeneration, represented by *w*_*C*5_. These results are consistent with prior observational studies based on ADNI and other longitudinal cohorts, which suggest that cognitive decline is driven primarily by high amyloid levels at earlier disease stages and by neurodegeneration at later stages^31^.

Sensitivity analysis also provides key insights in terms of personalized parameter selection. The paucity of longitudinal biomarker data and the relatively larger number of model parameters can easily lead to overfitting for personalized models. Based on the sensitivity analysis results, we chose the eight most sensitive population parameters as personalized parameters and set the rest of the parameters at the mean population parameter values. In this case, calibration of personalized parameters based on sparse longitudinal biomarker data for each patient avoids the overfitting issue and provides a high-precision personalized prediction for each subject, as outlined in Results section.

Limitations of this work include sampling bias. Because the ADNI dataset is a research cohort from academic clinics, only one-third of ADNI subjects agreed to provide CSF biomarkers. Thus we need to replicate these findings using data from more general practice settings in the future. Despite these limitations, this model advances our understanding of the complexity of AD biomarker pathophysiology over that of current biomarker models which have primarily been independent and ad hoc in nature, with inherent assumptions regarding the shape of individual biomarker trajectories. Our current approach is integrative and based on the cascade mechanism, yet without assumptions regarding the exact mathematical form of the individual biomarker models or the resulting shape of the biomarker trajectories. In the future, we intend to extend the current approach to the spatiotemporal domain by utilizing longitudinal imaging data to determine mechanisms driving the spread of pathology in time and space.

## Methods

We propose a pathophysiology and data-driven modeling approach to construct a causal model of AD clinical biomarkers. We construct a causal model from the serial clinical biomarker measures across 819 subjects from the ADNI-1 datasets with mild AD (N = 192), late mild cognitive impairment (LMCI, N = 398), and normal cognition (N = 229) (more details are shown in Table 4). We use PseudoIDs instead of RIDs to link across all clinical biomarker data belonging to a patient. The CSF proteins measured in ADNI are the following A-Beta 42 and Phosphorolated tau 181 (p-tau 181)^32,33^. These measures were obtained through serial spinal taps on subjects over approximately two-year intervals. Of note, A-Beta in the CSF goes down, and total and phosphorylated tau go up as the disease progresses. Hippocampal volume, a measure of neurodegeneration, was measured through volumetric analysis of serial MRI images obtained at approximately one-year intervals. It goes down as the disease progresses. Finally, cognitive decline was measured through a pencil-and-paper neuropsychological test, the thirteen-item Alzheimer s Disease Cognitive Assessment Scale (ADAS13). This measures function in several cognitive domains affected by AD, including memory, language, and praxis and is the de facto primary outcome measure in AD clinical trials. It goes up as the disease progresses.

**Table 4:** The characteristics of the ADNI-1 dataset in data-driven modeling.

	CN	LMCI	AD
N	229	398	192
Age	75.72 ± 4.86	74.52 ± 7.22	75.29 ± 7.41
M/F	119/110	257/141	101/91
MMSE	28.82 ± 1.78	25.54 ± 4.16	21.52 ± 4.59
ADAS-13	10.29 ± 6.44	21.71 ± 10.99	32.49 ± 10.42
CSF Ab42 (pg/ml)	201.74 ± 55.15	159.37 ± 51.53	139.79 ± 35.87
CSF Total Tau (pg/ml)	72.69 ± 31.69	104.65 ± 58.28	122.01 ± 58.30
CSF Ptau (pg/ml)	29.57 ± 16.10	38.96 ± 21.09	43.91 ± 20.97
Hipp Volume (ml)	7045.38 ± 971.27	6163.12 ± 1179.50	5488.95 ± 1132.57

**Fig. 5:**
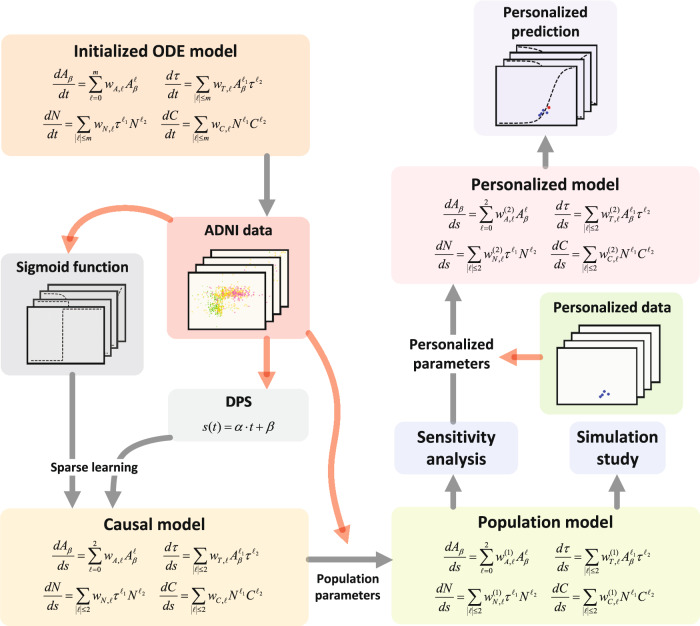
A flowchart of the pathophysiology and data-driven modeling approach. Given the initialized ODE model, a causal model is obtained by fitting the ADNI dataset and DPS model through sparse learning; secondly, the ADNI dataset is used to calibrate the population parameters in the causal model and obtain the population model; thirdly, a sensitivity analysis is applied to analyze the sensitivity of each population parameters and determine the sensitive personalized parameters, and a simulate study is conducted to validate the population model. Then, the personalized model is obtained by calibrating the sensitive personalized parameters with the use of personalized data. A prediction is made by the personalized model in the end.

After constructing and calibrating the population model with data across all ADNI subjects, we then personalize the parameters of the model using each patient’s longitudinal data to provide a personalized prediction of biomarker trajectories. The overall modeling approach is outlined in Fig. 5, and each step is elaborated in the following subsections.

### The data-driven causal model learning via ADNI dataset

Four AD biomarkers are key factors in AD diagnosis and monitoring of AD progression, and include amyloid-beta *A*_*β*_, tau *τ*, neuronal degeneration *N*, and cognitive decline *C*. Amyloid-beta is the main component of amyloid plaques and is considered to be an early event of the pathological cascade of AD. Amyloid production leads to downstream Tau phosphorylation causing the formation of neurofibrillary tangles and neuropil threads. Tau is a microtubule-associated protein, which is very common in neurons of the central nervous system. Both amyloid-beta and tau phosphorylation contribute to neuronal degeneration and cognitive decline.

To describe the cascade relationship among the above-mentioned four biomarkers of AD progression, we consider a canonical system of ODEs to describe their relations. The amyloid-dependent cascade is initiated by amyloid-beta pathology *A*_*β*_, and mediated via tau *τ*. Neuron degeneration *N* starts with the rise of tau *τ*, and in turn, leads to the initiation of cognitive decline *C*. According to the above description, we consider the causal model as the system of ODEs:

1 $$\left\{\begin{array}{lll}\frac{d{A}_{\beta }}{dt}=\mathop{\sum }\limits_{\ell = 0}^{m}{w}_{1,\ell }{\phi }_{\ell }({A}_{\beta });\frac{d\tau }{dt}=\mathop{\sum }\limits_{| {{{\boldsymbol{\ell }}}}| \le m}{w}_{2,{{{\boldsymbol{\ell }}}}}{\psi }_{{{{\boldsymbol{\ell }}}}}({A}_{\beta },\tau );\\ \frac{dN}{dt}=\mathop{\sum }\limits_{| {{{\boldsymbol{\ell }}}}| \le m}{w}_{3,{{{\boldsymbol{\ell }}}}}{\psi }_{{{{\boldsymbol{\ell }}}}}(\tau ,N);\frac{dC}{dt}=\mathop{\sum }\limits_{| {{{\boldsymbol{\ell }}}}| \le m}{w}_{4,{{{\boldsymbol{\ell }}}}}{\psi }_{{{{\boldsymbol{\ell }}}}}(N,C),\end{array}\right.$$

where ***ℓ*** = (*ℓ*_1_, *ℓ*_2_), ∣***ℓ***∣ = ∣*ℓ*_1_∣ + ∣*ℓ*_2_∣, and *m* is the degree of the model. We choose the polynomial basis function in the initialized ODE model, namely,

2 $${\phi }_{\ell }(x)={x}^{\ell },\,\,{\psi }_{{{{\boldsymbol{\ell }}}}}(x,y)={x}^{{\ell }_{1}}{y}^{{\ell }_{2}}.$$

We then learn the causal model parameters in (1) by using ADNI data. More specifically, we use CSF amyloid-beta 1-42 (*A*_*β*_), CSF total tau (*τ*), the ratio of hippocampal volume to whole-brain volume on MRI (*N*), and the Alzheimer’s Disease Assessment Scale-cognitive (*C*) to calibrate *A*_*β*_, *τ*, *N*, and *C*, respectively in the causal model. In order to denoise longitudinal data for different subjects, we applied a sigmoid interpolation for each biomarker. Moreover, because AD has a different time of onset and rate of progression for different subjects, we employ DPS^28^ to unify the time scale across subjects in the causal model.

### Disease progression scores

For different subjects in ADNI, the onset of disease and rate of progression are different within and among subject classes of CN, LMCI and AD. To fit the causal model for all subjects in the ADNI-1 study, we standardize the longitudinal measurement among patients by employing the DPS^28^. In particular, we define DPS *s*_*i*_(*t*) as a linear function of the patient’s age *t* for each patient:

3 $${s}_{i}(t)={\alpha }_{i}\cdot t+{\beta }_{i},$$

where *i* = 1, 2, ⋯ , *I* is the patient index, *α*_*i*_ is the rate of AD progression, and *β*_*i*_ is the age of AD onset.

### The sigmoid function fitting

We fit each biomarker data in ADNI to a sigmoid function. Specifically, each biomarker is parameterized by four parameters ${{{{\boldsymbol{\theta }}}}}_{k}={[{a}_{k},{b}_{k},{c}_{k},{d}_{k}]}^{T}$:

4 $${g}_{k}\left(s;{{{{\boldsymbol{\theta }}}}}_{k}\right)={a}_{k}{(1+{e}^{-{b}_{k}\left(s-{c}_{k}\right)})}^{-1}+{d}_{k},$$

where *a*_*k*_ is a magnitude scale of the function, *b*_*k*_ is a slope coefficient, and *c*_*k*_ and *d*_*k*_ determine function positions. Here we take *g*_1_(*s*) = *A*_*β*_(*s*), *g*_2_(*s*) = *τ*(*s*), *g*_3_(*s*) = *N*(*s*), *g*_4_(*s*) = *C*(*s*) and denote ${{{\boldsymbol{g}}}}={({g}_{1},{g}_{2},{g}_{3},{g}_{4})}^{T}$.

Next, we apply the sparse learning to reveal the causal model in (1) which is re-written as

$$\frac{d{{{\boldsymbol{x}}}}}{ds}=\mathop{\sum}\limits_{| \ell | \le m}{{{{\boldsymbol{\phi }}}}}_{\ell }({{{\boldsymbol{x}}}}){w}_{\ell },\,{{{\rm{where}}}}\,{{{\boldsymbol{x}}}}={({A}_{\beta },\tau ,N,C)}^{T}\in {R}^{4}.$$

By taking uniform grid points ${\{{s}_{i}\}}_{i = 1}^{M}$ on *s* ∈ [−10, 20], we denote

$${D}_{i}=[{\phi }_{{\ell }_{1}}({{{\boldsymbol{g}}}}({s}_{i})),\cdots \,,{\phi }_{{\ell }_{n}}({{{\boldsymbol{g}}}}({s}_{i}))]\,and\,{b}_{i}=\frac{d({{{\boldsymbol{g}}}}({s}_{i}))}{ds},$$

where *ℓ*_1_, ⋯ , *ℓ*_*n*_ are in the set of ∣*ℓ*∣ ≤ *m*. By expanding

$$D=\left(\begin{array}{l}{D}_{1}\\ \vdots \\ {D}_{M}\end{array}\right){{{\rm{and}}}}\,b=\left(\begin{array}{l}{b}_{1}\\ \vdots \\ {b}_{M}\end{array}\right),$$

we learn the causal model via the following Lasso regression, namely,

5 $$\mathop{\min }\limits_{w}\parallel Dw-b{\parallel }_{2}^{2}+\lambda \parallel w{\parallel }_{1},$$

where ∥*w*∥_1_ enforces the sparsity.

Here we keep the polynomial degrees among all the variables in the causal model be consistent and choose *m* = 4 with *λ* = 10^−7^ in (5). By performing Lasso, we find the result is consistent with the causal model when *m* = 2 but different from the one with *m* = 1, which indicates the optimal choice of the causal model is *m* = 2. Then the general causal model of ODEs describing the progression of AD biomarkers is summarized below (All rights to the in-silico model belong to the authors and it cannot be used for any commercial purpose without permission):

6 $$\left\{\begin{array}{l}\frac{d{A}_{\beta }}{ds}={w}_{A0}+{w}_{A1}{A}_{\beta }+{w}_{A2}{A}_{\beta }^{2};\\ \frac{d\tau }{ds}={w}_{T0}+{w}_{T1}\tau +{w}_{T2}{\tau }^{2}+{w}_{T3}{A}_{\beta }+{w}_{T4}{A}_{\beta }^{2}+{w}_{T5}{A}_{\beta }\tau ;\\ \frac{dN}{ds}={w}_{N0}+{w}_{N1}N+{w}_{N2}{N}^{2}+{w}_{N3}\tau +{w}_{N4}{\tau }^{2}+{w}_{N5}\tau N;\\ \frac{dC}{ds}={w}_{C0}+{w}_{C1}C+{w}_{C2}{C}^{2}+{w}_{C3}N+{w}_{C4}{N}^{2}+{w}_{C5}NC,\end{array}\right.$$

with an initial condition *A*_*β*_(−10) = *y*_0_ and *τ*(−10) = *N*(−10) = *C*(−10) = 0, where *y*_0_ is also a parameter that we consider as a small positive value to initiate the cascade.

### Population model calibration

First, we calibrate the learned causal model by using the ADNI dataset and rewrite (6) as the following population model

7 $$\left\{\begin{array}{lll}\frac{d{A}_{\beta }}{ds}=\mathop{\sum }\limits_{\ell = 0}^{2}{w}_{A,\ell }^{(1)}{A}_{\beta }^{\ell },\frac{d\tau }{ds}=\mathop {\sum}\limits_{| \ell | \le 2}{w}_{T,\ell }^{(1)}{A}_{\beta }^{{\ell }_{1}}{\tau }^{{\ell }_{2}},\\ \frac{dN}{ds}=\mathop {\sum}\limits_{| \ell | \le 2}{w}_{N,\ell }^{(1)}{\tau }_{\rho }^{{\ell }_{1}}{N}^{{\ell }_{2}},\frac{dC}{ds}=\mathop {\sum}\limits_{| \ell | \le 2}{w}_{C,\ell }^{(1)}{N}^{{\ell }_{1}}{C}^{{\ell }_{2}},\end{array}\right.$$

where ${{{\boldsymbol{w}}}}=\{{w}_{A,\ell }^{(1)},{w}_{T,\ell }^{(1)},{w}_{N,\ell }^{(1)},{w}_{C,\ell }^{(1)}\}$ denote the population parameters. We also denote *f*_1_(*s*) = *A*_*β*_(*s*), *f*_2_(*s*) = *τ*(*s*), *f*_3_(*s*) = *N*(*s*), *and* *f*_4_(*s*) = *C*(*s*) with the initial conditions *f*_1_(−10) = *y*_0_, *f*_2_(−10) = *f*_3_(−10) = *f*_4_(−10) = 0. Then the population parameters are calibrated based on the ADNI dataset by minimizing the sum of squared differences between the data and the solution of the causal model, namely

8 $$\mathop{\min }\limits_{{{{\boldsymbol{{w}}}_{k}}}}\mathop{\sum}\limits_{(i,j)\in {{{{\mathcal{I}}}}}_{k}}{\left({y}_{ijk}-{f}_{k}\left({\alpha }_{i}{t}_{ij}+{\beta }_{i};{{{{\boldsymbol{w}}}}}_{k}\right)\right)}^{2},\,\,(i,j,k)\in {{{\mathcal{I}}}}$$

where *y*_*ijk*_ is the *k*-th biomarker data for *i*-th patient at *j*-th visit and ${{{{\mathcal{I}}}}}_{k}$ is the set of (*i*, *j*) for *k*-th biomarker.

Since the biomarkers for each patient will generally increases or decreases monotonically, we consider fitting DPS as a least square linear regression problem, namely,

9 $$\mathop{\min }\limits_{{\alpha }_{i},{\beta }_{i}}\mathop{\sum}\limits_{(j,k)\in {{{{\mathcal{I}}}}}_{i}}\frac{1}{{\sigma }_{k}}{\left({y}_{ijk}-{f}_{k}\left({\alpha }_{i}{t}_{ij}+{\beta }_{i};{{{{\boldsymbol{w}}}}}_{k}\right)\right)}^{2},$$

where ${{{{\mathcal{I}}}}}_{i}$ is set of (*j*, *k*) for *i*-th patient and *σ*_*k*_ is the sum of squared error with respect to biomarker *k*, namely,

10 $${\sigma }_{k}=\frac{1}{| {{{{\mathcal{I}}}}}_{k}-2I-4| }\mathop{\sum}\limits_{(i,j)\in {{{{\mathcal{I}}}}}_{k}}{\left({y}_{ijk}-{f}_{k}\left({\alpha }_{i}{t}_{ij}+{\beta }_{i};{{{{\boldsymbol{w}}}}}_{k}\right)\right)}^{2}.$$

The detailed procedure to fit the parameters is shown in Algorithm 1. The optimization solver employs the Levenberg-Marquardt method^34^, which can avoid getting stuck in a local minimum.

### Sensitivity analysis

We assume that the parameters in the population model, ${{{{\boldsymbol{w}}}}}^{(1)}=[{w}_{A0}^{(1)},\,{w}_{A1}^{(1)},\,\cdots \,,\,{w}_{m}^{(1)},\,\cdots \,,\,{w}_{C4}^{(1)},\,{w}_{C5}^{(1)}]\in {{\mathbb{R}}}^{21}$, are independent and identically distributed inputs, where *m* is the index of inputs. For sensitivity analysis, we omit the superscript of the parameters later for simplicity. The range of each input is 90–110% of their values shown in Table 1.

Then we perform Sobol sensitivity analysis, which is also called variance-based sensitivity analysis and is developed from the analysis of variance. As a global sensitivity analysis method, it analyzes the effects of each input by decomposing the variance of the output of the population model into fractions attributed to the inputs. In this paper, we perform both the first-order and second-order sensitivity analyses to the parameters. In particular, the first-order sensitivity index measures the attribution to the variance of the output considering only one input, which is calculated by:

11 $${{{{\rm{S}}}}}_{m}(y)=\frac{{{{{\rm{Var}}}}}_{{w}_{m}}\left[{{{{\rm{E}}}}}_{{{{{\rm{w}}}}}_{ \sim {{{\rm{m}}}}}}(y| {w}_{m})\right]}{{{{\rm{Var}}}}(y)},$$

where ${w}_{ \sim m}=\left[{w}_{A1},\,\cdots \,,\,{w}_{m-1},\,{w}_{m+1},\,\cdots \,,\,{w}_{C5}\right]$ includes all inputs except *w*_*m*_. Next, the second order sensitivity with respect to *m* and *n* is measured by sum of attributing the variance of the output considering their first order effects and the second-order interaction between inputs *m* and *n*:

12 $${{{{\rm{S}}}}}_{(m,n)}(y)={{{{\rm{S}}}}}_{m}(y)+{{{{\rm{S}}}}}_{n}(y)+\frac{{{{{\rm{Var}}}}}_{({w}_{m},{w}_{n})}\left[{{{{\rm{E}}}}}_{{{{{\rm{w}}}}}_{ \sim {{{\rm{m}}}},{{{\rm{n}}}}}}(y| {w}_{m},{w}_{n})\right]}{{{{\rm{Var}}}}(y)}.$$

Then we measure the total-order sensitivity index, which is calculated by attributing the variance of the output considering both the first-order effect, second-order effect, and other higher-order ones.

13 $${{{{\rm{S}}}}}_{T,m}(y)=1-\frac{{{{{\rm{Var}}}}}_{{{{\rm{{w}}}_{ \sim m}}}}\left[{{{{\rm{E}}}}}_{{w}_{m}}(y| {{{\rm{{w}}}_{ \sim m}}})\right]}{{{{\rm{Var}}}}(y)}.$$

When the sensitivity value is positive, the corresponding parameter is positively correlated with the model output. If the value is negative, they are negatively correlated. The absolute value of parameter sensitivities represents the degree of influence on the model output. If the sensitivity value is closer to 0, changing this parameter will have less influence on the model output. Based on the sensitivity values and the number of biomarker measurements, we determine the personalized parameters to fit the longitudinal data points for each patient and keep the remaining parameters the same as the population parameter values. This can avoid overfitting when providing the personalized prediction for each subject.

### Reporting summary

Further information on research design is available in the Nature Research Reporting Summary linked to this article.

## Supplementary information

Supplementary Information

Reporting Summary

## Data Availability

Access to the ADNI dataset is publicly available via http://adni.loni.usc.edu^35^.

## Code availability

The sensitivity analysis code is available at http://salib.readthedocs.io/en/latest/. The simulation study code is available at https://www.pymc.io/welcome. The non-linear optimizer can be found in https://github.com/jjhartmann/Levenberg-Marquardt. Codes for Algorithms 1 and 2 are included in the Supplementary Information.

## References

[1] Cortes-Canteli M, Iadecola C (2020). Alzheimer’s disease and vascular aging: Jacc focus seminar. J. Am. College Cardiol..

[2] Batool A, Kamal MA, Rizvi S, Rashid S (2018). Topical discoveries on multi-target approach to manage alzheimer’s disease. Curr Drug Metab..

[3] Bertram L, McQueen MB, Mullin K, Blacker D, Tanzi RE (2007). Systematic meta-analyses of Alzheimer disease genetic association studies: the AlzGene database. Nat. Genet.

[4] Lane CA, Hardy J, Schott JM (2018). Alzheimer's disease. Eur. J. Neurol..

[5] Aliev G (2019). Alzheimer’s disease–future therapy based on dendrimers. Curr. Neuropharmacol..

[6] Milne R (2018). At, with and beyond risk: expectations of living with the possibility of future dementia. Soc. Health Illness.

[7] Sperling RA (2011). Toward defining the preclinical stages of Alzheimer’s disease: recommendations from the National Institute on Aging-Alzheimer’s Association workgroups on diagnostic guidelines for Alzheimer’s disease. Alzheimers Dement.

[8] Jack CR (2018). NIA-AA research framework: toward a biological definition of Alzheimer’s disease. Alzheimers Dement.

[9] Hao W, Friedman A (2016). Mathematical model on Alzheimer’s disease. BMC Syst Biol.

[10] Petrella, J. R., Hao, W., Rao, A. & Doraiswamy, P. M. Computational causal modeling of the dynamic biomarker cascade in Alzheimer’s disease. *Comput. Math. Methods Med.* **2019**, 10.1155/2019/6216530 (2019). 10.1155/2019/6216530 PMC6378032 30863455

[11] Jack CR, Holtzman DM (2013). Biomarker modeling of Alzheimer’s disease. Neuron.

[12] Abeysinghe AADT, Deshapriya RDUS, Udawatte C (2020). Alzheimer’s disease; a review of the pathophysiological basis and therapeutic interventions. Life Sci.

[13] Guo T, Korman D, Baker SL, Landau SM, Jagust WJ (2021). Longitudinal cognitive and biomarker measurements support a unidirectional pathway in Alzheimer’s disease pathophysiology. Biol. Psychiatry.

[14] Myszczynska MA (2020). Applications of machine learning to diagnosis and treatment of neurodegenerative diseases. Nat. Rev. Neurol..

[15] Iturria-Medina Y, Carbonell FM, Sotero RC, Chouinard-Decorte F, Evans AC (2017). Multifactorial causal model of brain (dis)organization and therapeutic intervention: Application to Alzheimer’s disease. Neuroimage.

[16] Friedman A, Hao W (2018). The role of exosomes in pancreatic cancer microenvironment. Bull. Math. Biol..

[17] Budithi, A., Su, S., Kirshtein, A. & Shahriyari, L. Data driven mathematical model of FOLFIRI treatment for colon cancer. *Cancers.* **13**,10.3390/cancers13112632 (2021). 10.3390/cancers13112632 PMC8198096 34071939

[18] Hao W (2017). A mathematical model of aortic aneurysm formation. PLoS One.

[19] Friedman, A. & Hao, W. A mathematical model of atherosclerosis with reverse cholesterol transport and associated risk factors. *Bull. Math. Biol*. 77, 758-781 (2015). 10.1007/s11538-014-0010-3 25205457

[20] Wang X (2018). A bayesian framework for generalized linear mixed modeling identifies new candidate loci for late-onset alzheimer’s disease. Genetics.

[21] Sun N (2019). Multi-modal latent factor exploration of atrophy, cognitive and tau heterogeneity in alzheimer’s disease. Neuroimage.

[22] Schäfer, A. et al. Bayesian physics-based modeling of tau propagation in alzheimer’s disease. *Front. Physiol.* 1081, 10.3389/fphys.2021.702975 (2021). 10.3389/fphys.2021.702975 PMC8322942 34335308

[23] Iddi S (2018). Estimating the evolution of disease in the parkinson’s progression markers initiative. Neurodegenerative Dis..

[24] Iddi S (2019). Predicting the course of alzheimer’s progression. Brain Informatics.

[25] Li D (2019). The relative efficiency of time-to-progression and continuous measures of cognition in presymptomatic alzheimer’s disease. Alzheimer’s & Dement..

[26] Li D, Iddi S, Thompson WK, Donohue MC, Initiative ADN (2019). Bayesian latent time joint mixed effect models for multicohort longitudinal data. Stat. Methods Med. Res..

[27] Marinescu RV (2020). Predicting alzheimer’s disease progression: Results from the tadpole challenge: Neuroimaging: Neuroimaging predictors of cognitive decline. Alzheimer’s Dement..

[28] Jedynak BM (2012). A computational neurodegenerative disease progression score: method and results with the alzheimer’s disease neuroimaging initiative cohort. Neuroimage.

[29] Sobol IM (2001). Global sensitivity indices for nonlinear mathematical models and their monte carlo estimates. Math. Comput. Simul..

[30] Zhang S, Ponce J, Zhang Z, Lin G, Karniadakis G (2021). An integrated framework for building trustworthy data-driven epidemiological models: Application to the covid-19 outbreak in new york city. PLOS Comput. Biol..

[31] Jack CR (2009). Serial PIB and MRI in normal, mild cognitive impairment and Alzheimer’s disease: implications for sequence of pathological events in Alzheimer’s disease. Brain.

[32] Shaw LM (2011). Qualification of the analytical and clinical performance of CSF biomarker analyses in ADNI. Acta Neuropathol.

[33] Shaw LM (2008). PENN biomarker core of the Alzheimer’s disease Neuroimaging Initiative. Neurosignals.

[34] Levenberg K (1944). A method for the solution of certain non-linear problems in least squares. Quart. Appl. Math..

[35] Weiner MW (2013). The alzheimer’s disease neuroimaging initiative: a review of papers published since its inception. Alzheimer’s Dement..

