# Supplementary material for: Data-driven causal model discovery and personalized prediction in Alzheimer's disease
Source: NPJ Digit Med. 2022 Sep 8;5:137. doi: 10.1038/s41746-022-00632-7 (PMC9458727; doi:10.1038/s41746-022-00632-7)
Supplement: Supplementary file 1 — Supplementary Information [file 41746_2022_632_MOESM1_ESM.pdf]

Supplementary Information:
Data-driven AD Model

Haoyang Zheng, Jeffrey R. Petrella, P. Murali Doraiswamy,
Guang Lin, Wenrui Hao,
and for the Alzheimer's Disease Neuroimaging Initiative

June 14, 2022

1 Simulation Study

We run the simulation study for the population parameter by using MCMC. More specifically, we use MCMC with different sample points are drawn based on the normal distribution for each population parameter and evaluate three different quantities:

1) total bias (TB) calculated as TB $= \frac{\sum_{i=1}^{N}(\hat{w}_i - \bar{w})}{N}$;

2) MSE calculated by MSE $= \frac{\sum_{i=1}^{N}(\hat{w}_i - \bar{w})^2}{N}$; and

3) the 95% confidence interval defined by

$$C_{95} = \sum_{i=1}^{N} \frac{\mathbb{1}_{\{\hat{w}_i \in [\bar{w}-1.96\sigma, \bar{w}+1.96\sigma]\}}}{N}.$$

Here N is the number of MCMC sample points, $\hat{w}_i$ denotes each sample, $\bar{w}$ is the prior mean for each parameter, and σ is the posterior standard deviation of the parameter $\bar{w}$.

Each population parameter is drawn from a normal distribution with the mean shown in Table 2 and the variance is 0.1. The simulation is run with four chains (each chain has 1,000 posterior samples, $N = 4000$) by using the No-U-Turn Sampler (NUTS) algorithm. We summarize three-parameter quantities in Table 1. MCMC simulation results for each biomarker are shown in Figs. 1-4.

Table 1: Simulation study results by using N = 4000 MCMC sample points for the population parameters.

Weights		Population model		
		TB	MSE	C_{95}
A_β	w_{A1}	0.015	0.012	0.956
	w_{A2}	0.015	0.016	0.958
τ	w_{T1}	0.078	0.012	0.944
	w_{T2}	-0.047	0.011	0.931
	w_{T3}	0.002	<0.001	0.975
	w_{T4}	-0.020	0.008	0.931
	w_{T5}	-0.040	0.010	0.946
N	w_{N1}	0.013	0.002	0.943
	w_{N2}	0.008	0.007	0.955
	w_{N3}	-0.010	0.008	0.953
	w_{N4}	0.005	0.007	0.954
	w_{N5}	-0.007	0.008	0.954
C	w_{C1}	-0.020	0.010	0.957
	w_{C2}	0.005	0.010	0.947
	w_{C3}	-0.008	0.004	0.958
	w_{C4}	0.003	0.006	0.952
	w_{C5}	0.006	0.010	0.949

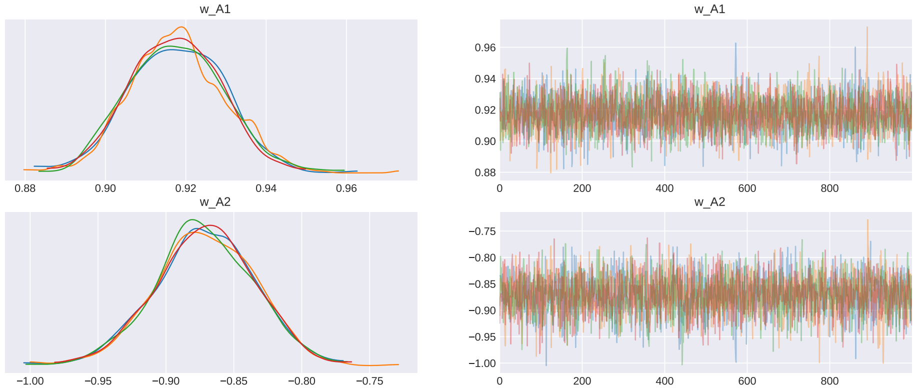

Figure 1: MCMC simulation results of Abeta in the population model. **Left:** posterior distribution histogram of each population parameter (using kernel density estimation). The histogram with different colors represents different chains starting from different points (with a small noise). **Right:** the samples of the Markov chain plotted in sequential order. Lines of different colors represent different chains running in parallel.

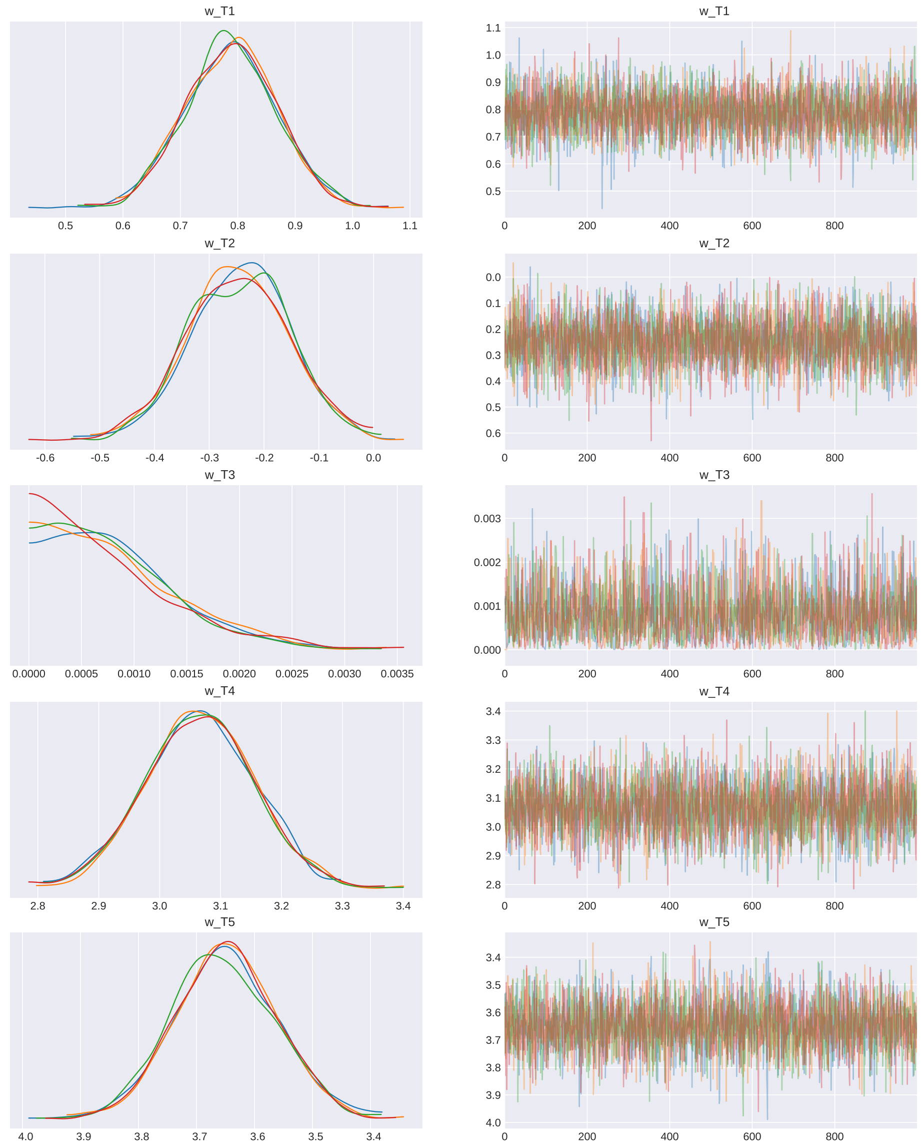

Figure 2: MCMC simulation results of tau in the population model. **Left:** posterior distribution histogram of each population parameter (using kernel density estimation). The histogram with different colors represents different chains starting from different points (with a small noise). **Right:** the samples of the Markov chain plotted in sequential order.

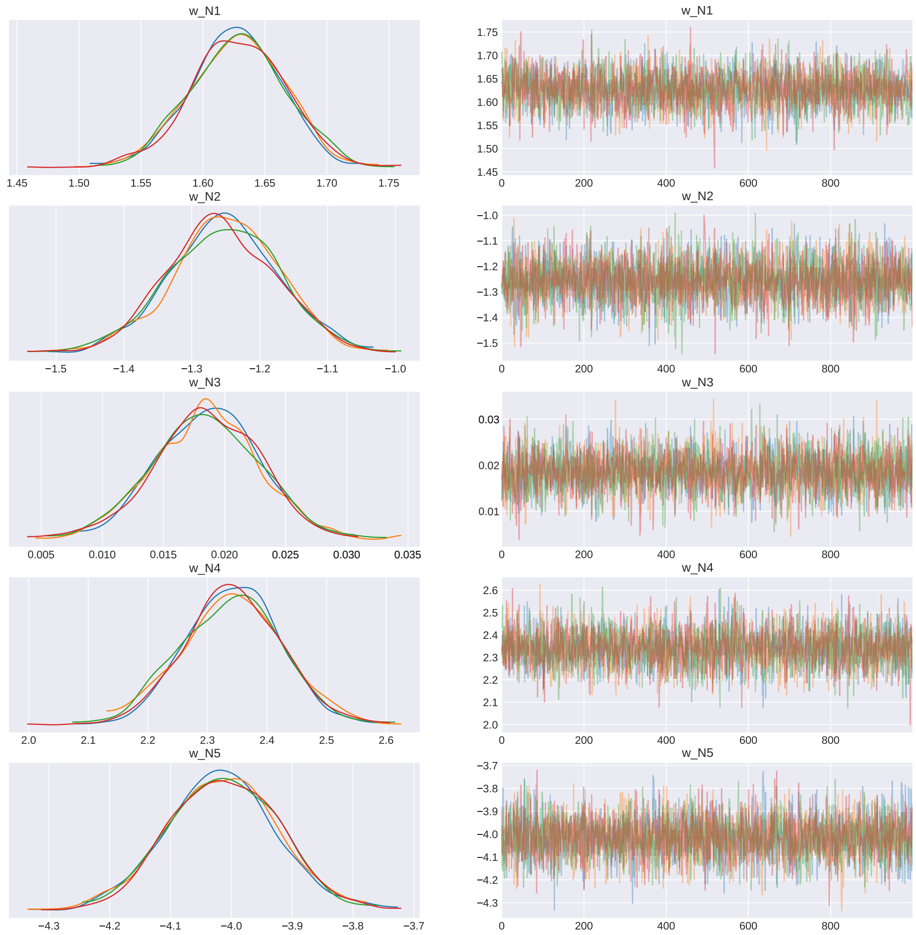

Figure 3: MCMC simulation results of hippocampal volume in the population model. **Left:** posterior distribution histogram of each population parameter (using kernel density estimation). The histogram with different colors represents different chains starting from different points (with a small noise). **Right:** the samples of the Markov chain plotted in sequential order.

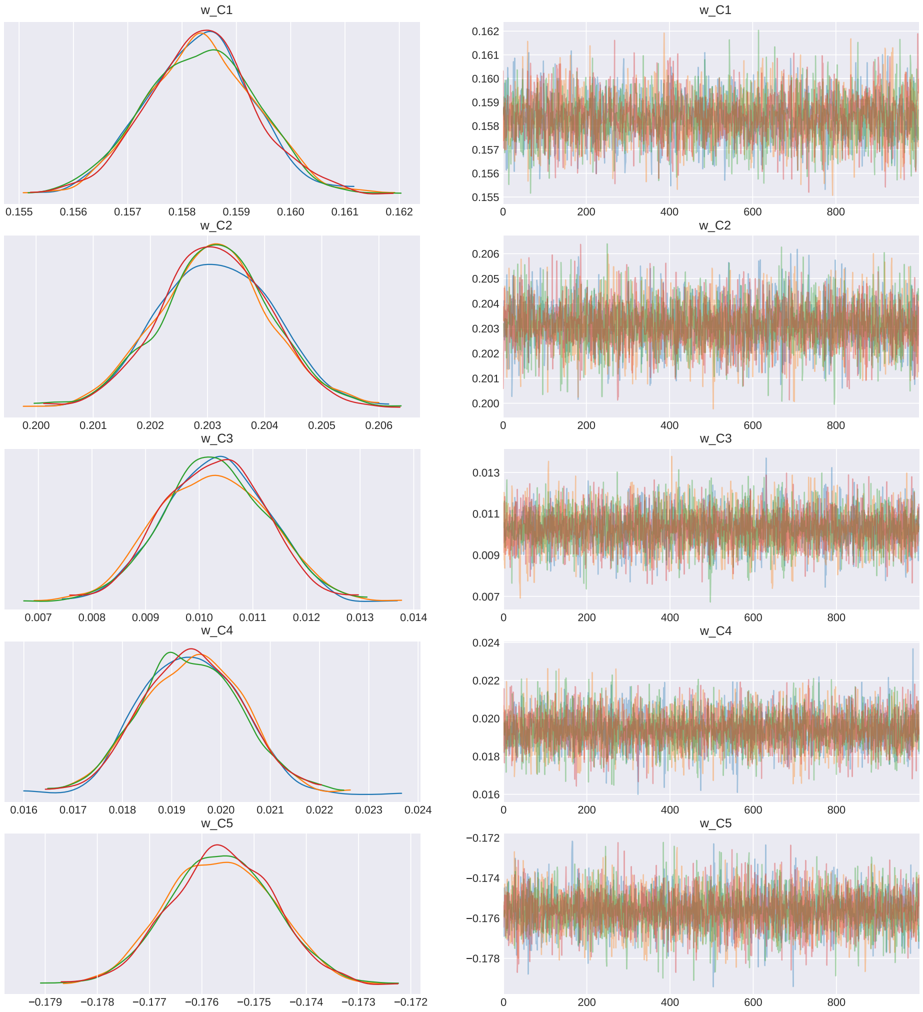

Figure 4: MCMC simulation results of ADAS13 in the population model. **Left:** posterior distribution histogram of each population parameter (using kernel density estimation). The histogram with different colors represents different chains starting from different points (with a small noise). **Right:** the samples of the Markov chain plotted in sequential order.

2 Diagnostic Plots for Figs. 5(b) & 5(c)

We use the Normal Quantile-Quantile (Q-Q) plot to test if residuals are normally distributed for each biomarker. Based on Figs. 5 and 6, we clearly see that the residuals follow a straight line well and therefore are normally distributed.

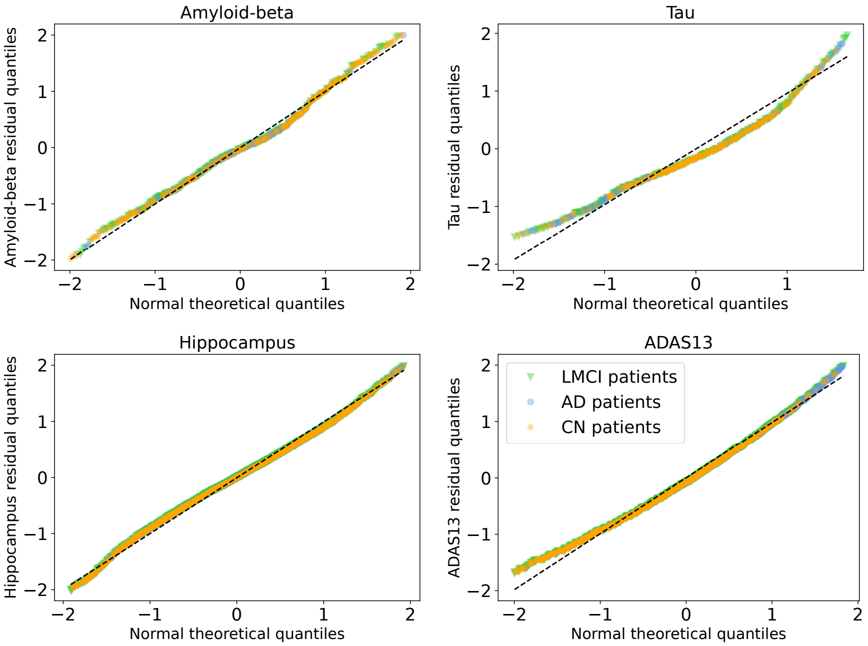

Figure 5: Normal Q-Q plots for Fig. 5(b). The x-axes is the normal distribution while the y-axes is the residual for each biomarker inside the 95% confidence interval.

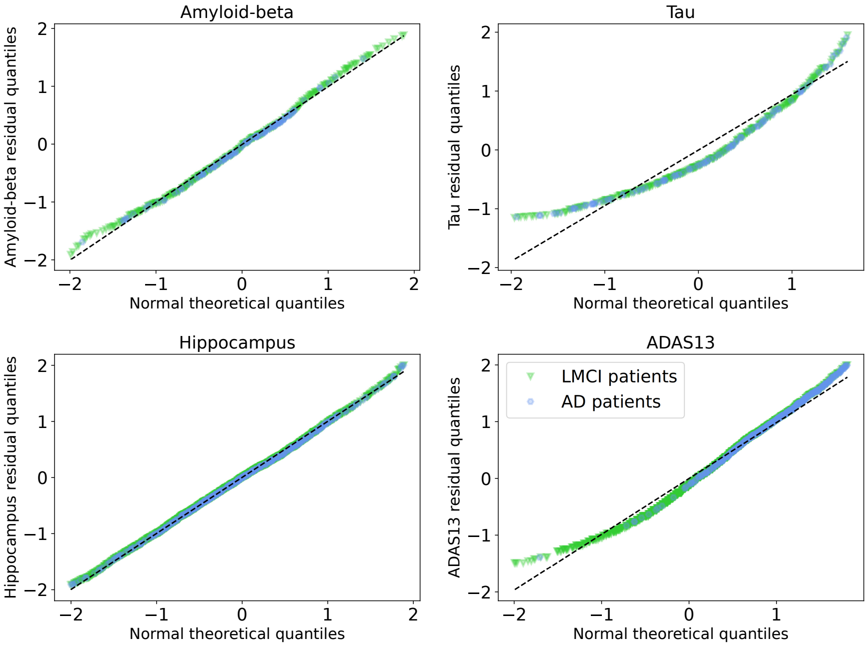

Figure 6: Normal Q-Q plots for Fig. 5(c). The quantiles only include the ones inside the 95% confidence interval.

3 Supplemental Code 1

```python
import numpy as np
from Function.LogisticRegression import fit_population, dps_error, population_error
from Function.LevenbergMarquardt.LMA import LevenbergMarquardt
np.random.seed(0)


def population(bio_data, omega, num_iter=1000):
    """ Algorithm 1: Population model calibration algorithm.
        :param  bio_data:   subject visit ages and the corresponding
                            recorded biomarkers. Saved in shape [j, k+1,
                            i], where j is the maximum visit number, k is
                            the number of recorded biomarkers ("+1" for the
                            saved ages), and i is the number of subject.
        :param  omega:      initialized population parameters. Saved in
                            shape [m, k], where m is the maximum number of
                            parameters for each biomarker.
        :param  num_iter:   number of iterations (L)
        :return  alpha:     rate of progression
        :return  beta:      onset age
        :return  omega:     population parameters
        """

    num_sub = bio_data.shape[2]
    bio_idx = [_ for _ in range(bio_data.shape[1] - 1)]
    alpha = np.zeros(num_sub)
    beta = np.zeros(num_sub)
    sigma = np.zeros((4, 1))

    # choose the initial DPS parameters
    for i in range(num_sub):
        max_ = np.max(bio_data[:, 0, i][~np.isnan(bio_data[:, 0, i])])
        min_ = np.min(bio_data[:, 0, i][~np.isnan(bio_data[:, 0, i])])
        alpha[i] = np.random.uniform(low=0, high=4, size=1)
        beta[i] = np.random.uniform(low=-10 - alpha[i] * min_, high=20 - alpha[i] * max_, size=1)

    for i in range(num_iter):

        # update DPS
        for i in range(num_sub):
            try:
                dps = np.hstack((
                    dps, bio_data[:, 0, i][~np.isnan(bio_data[:, 0, i])] * alpha[i] + beta[i]))
                biomarker = np.concatenate((
                    biomarker, bio_data[:, [_ + 1 for _ in bio_idx], i][~np.isnan(bio_data[:, 0, i])]),
                    axis=0)
            except NameError:
                dps = bio_data[:, 0, i][~np.isnan(bio_data[:, 0, i])] * alpha[i] + beta[i]
                biomarker = bio_data[:, [_ + 1 for _ in bio_idx], i][~np.isnan(bio_data[:, 0, i])]

```

```python
        # update population parameters
        for k in range(len(bio_idx)):
            index_exist = np.where(~np.isnan(biomarker[:, k]))
            dps_exist = dps[index_exist]
            if k == 0:
                bio_exist = biomarker[index_exist][:, k]
            else:
                bio_exist = biomarker[index_exist][:, k-1:k+1]

            # error = population_error((omega[k]), (dps_exist,
    bio_exist))
            alpha[i], beta[i] = LevenbergMarquardt(
                seed_params=omega[k],
                args=(dps_exist, bio_exist),
                error_function=population_error)[1]

            pred = fit_population(dps_exist, omega[k])
            sigma[k] = np.mean(np.square(pred - bio_exist))

        # update DPS parameters
        for i in range(num_sub):

            Y = bio_data[:, [_ + 1 for _ in bio_idx], i][~np.isnan(
    bio_data[:, 0, i])]
            X = bio_data[:, 0, i][~np.isnan(bio_data[:, 0, i])]

            # error = dps_error((alpha[i], beta[i]), (X, Y, omega,
    sigma))
            alpha[i], beta[i] = LevenbergMarquardt(
                seed_params=np.hstack((alpha[i], beta[i])),
                args=(X, Y, omega, sigma),
                error_function=dps_error)[1]

    return alpha, beta, omega
```

Listing 1: Population model calibration algorithm.

4 Supplemental Code 2

```python
from scipy.optimize import curve_fit
import ode_fit1, ode_fit2, pred_func
from scipy.integrate import odeint
import numpy as np


def personalized(bio_data, omega, alpha, beta, pid, par_idx, initial
    =6.35*1e-6):
    """ Algorithm 2: Personalized model calibration algorithm.
        :param  bio_data:   subject visit ages and the corresponding
                          recorded biomarkers.
        :param  omega:      population parameters.
        :param  alpha:      rate of progression
        :param  beta:       onset age
        :param  pid:        subject index
        :param  par_idx:    parameter index (boolean, list: 4). The
```

```
                          boolean values are selected by sensitivity
                          analysis, where True value means sensitive
                          parameter, and False means insensitive
                          parameter. Same shape as omega.
        :param  initial:    initial condition
        :return  acc:       prediction accuracy for each biomarker
        """

    num_sub = bio_data.shape[2]
    bio_idx = [_ for _ in range(bio_data.shape[1] - 1)]
    dps = np.zeros(0)
    id_pat = np.zeros(0)
    bio = np.zeros((0, bio_data.shape[1]-1))

    # select subject data. See "personalized model and biomarker prediction" part for detail.
    for i in range(num_sub):
        # iterate all records with biomarkers
        for j in range((~np.isnan(bio_data[:, 0, i])).sum()):
            # drop data if not all biomarkers are available
            if np.isnan(bio_data[j, :, i]).sum() != 1:
                bio_data[j, :, i].fill(np.nan)

        if (~np.isnan(bio_data[:, 0, i])).sum() > 3:
            # check whether the subject visit more than 3 times
            subject_AD = np.concatenate((subject_AD, bio_data[:, :, i:i + 1]), axis=2)
            dps = np.hstack((dps, bio_data[:, 0, i][~np.isnan(bio_data[:, 0, i])] * alpha[i] + beta[i]))
            # record the biomarkers belong to which subject
            id_pat = np.hstack((id_pat, i * np.ones(((~np.isnan(bio_data[:, 0, i])).sum()))))
            bio = np.concatenate(
                (bio, bio_data[:, [_ + 1 for _ in bio_idx], i][~np.isnan(bio_data[:, 0, i])]), axis=0)

    # locate subject data
    for m in range(len(dps)):
        if id_pat[i] == pid:
            idx_extract = np.concatenate((idx_extract, np.array(m).reshape(-1)), axis=0)

    # some subjects did not record following their visit time. Reorganize the order based on visiting time to avoid errors.
    c = zip(dps, bio.reshape(-1, 4))
    bio_sort = sorted(c)
    dps, bio = zip(*bio_sort)

    dps = np.array(dps)
    bio = np.array(bio)

    # training data
    dps_train = dps[idx_extract.astype(int)].reshape(-1)[:-1]
    bio_train = bio[idx_extract.astype(int)].reshape(-1)[:-1]

    # testing data
    dps_test = dps[idx_extract.astype(int)].reshape(-1)[-1]
```

```
    bio_test = bio[idx_extract.astype(int)].reshape(-1)[-1]

    # update personalized parameters
    omega_personal = 1 * omega
    for k in range(len(bio_idx)):
        if k == 0:
            sense_idx = np.where(par_idx[k])
            dps_ = np.concatenate((omega[k, ~sense_idx], dps_train))
            popt, _ = curve_fit(ode_fit1, dps_, bio_train[:, k], omega[k, sense_idx])
        else:
            sense_idx = np.where(par_idx[k])
            dps_ = np.concatenate((omega[k, ~sense_idx], dps_))
            popt, _ = curve_fit(ode_fit2, dps_, bio_train[:, k - 1:k + 1], omega[k, sense_idx])

        # update personalized parameters
        for i in range(len(sense_idx)):
            omega_personal[k, sense_idx[i]] = popt[i]

    tt = np.linspace(-10, 20, 301)
    ode_pred = odeint(pred_func, [initial, 0, 0, 0], tt, args=omega_personal)

    # make prediction
    bio_name = ["A-beta", "Tau", "Hippocampus", "ADAS13"]
    acc = np.zeros(4)
    for k in range(len(bio_idx)):
        idx_predic = np.where(np.around(tt, decimals=1) == np.around(dps_test, decimals=1))
        acc[k] = np.abs(ode_pred[idx_predic, k] - bio_test[k]) / bio_test[k]
        print("Prediction accuracy of " + bio_name[k] + " is %.4f." % acc[k])

    return acc
```

Listing 2: Personalized model calibration algorithm.
